# Ancient nitrogenases are ATP dependent

**DOI:** 10.1128/mbio.01271-24

**Published:** 2024-06-13

**Authors:** Derek F. Harris, Holly R. Rucker, Amanda K. Garcia, Zhi-Yong Yang, Scott D. Chang, Hannah Feinsilber, Betül Kaçar, Lance C. Seefeldt

**Affiliations:** 1 Department of Chemistry and Biochemistry, Utah State University, Logan, Utah, USA; 2 Department of Bacteriology, University of Wisconsin–Madison, Madison, Wisconsin, USA; University of Washington School of Medicine, Seattle, Washington, USA

**Keywords:** nitrogen fixation, ancestral sequence reconstruction, energy

## Abstract

Life depends on a conserved set of chemical energy currencies that are relics of early biochemistry. One of these is ATP, a molecule that, when paired with a divalent metal ion such as Mg^2+^, can be hydrolyzed to support numerous cellular and molecular processes. Despite its centrality to extant biochemistry, it is unclear whether ATP supported the function of ancient enzymes. We investigate the evolutionary necessity of ATP by experimentally reconstructing an ancestral variant of the N_2_-reducing enzyme nitrogenase. The Proterozoic ancestor is predicted to be ~540–2,300 million years old, post-dating the Great Oxidation Event. Growth rates under nitrogen-fixing conditions are ~80% of those of wild type in *Azotobacter vinelandii*. In the extant enzyme, the hydrolysis of two MgATP is coupled to electron transfer to support substrate reduction. The ancestor has a strict requirement for ATP with no other nucleotide triphosphate analogs (GTP, ITP, and UTP) supporting activity. Alternative divalent metal ions (Fe^2+^, Co^2+^, and Mn^2+^) support activity with ATP but with diminished activities compared to Mg^2+^, similar to the extant enzyme. Additionally, it is shown that the ancestor has an identical efficiency in ATP hydrolyzed per electron transferred to the extant of two. Our results provide direct laboratory evidence of ATP usage by an ancient enzyme.

**IMPORTANCE:**

Life depends on energy-carrying molecules to power many sustaining processes. There is evidence that these molecules may predate the rise of life on Earth, but how and when these dependencies formed is unknown. The resurrection of ancient enzymes provides a unique tool to probe the enzyme’s function and usage of energy-carrying molecules, shedding light on their biochemical origins. Through experimental reconstruction, this research investigates the ancestral dependence of a nitrogen-fixing enzyme on the energy carrier ATP, a requirement for function in the modern enzyme. We show that the resurrected ancestor does not have generalist nucleotide specificity. Rather, the ancestor has a strict requirement for ATP, like the modern enzyme, with similar function and efficiency. The findings elucidate the early-evolved necessity of energy-yielding molecules, delineating their role in ancient biochemical processes. Ultimately, these insights contribute to unraveling the intricate tapestry of evolutionary biology and the origins of life-sustaining dependencies.

## INTRODUCTION

The use of ATP as an energy carrier is a ubiquitous feature of life. Energy transfer reactions that depend on the hydrolysis of ATP to ADP (typically complexed with Mg^2+^) and Pi are shared by all modern organisms. This universality and its role in RNA synthesis suggest that ATP is a remnant of ancient, possibly prebiotic biochemistry (1, 2). Indeed, extant enzymes that require or synthesize ATP are similarly widespread across the tree of life and are proposed to have originated prior to the last universal common ancestor (3–5).

The role of ATP in biology is not only ancient but, over more than 3.5 billion years of evolution (6), it emerged as the principal energy currency of life (1, 7). It is not known when or why the predominance of ATP relative to other nucleoside triphosphates, like GTP, was established as the energy carrier. Several lines of evidence suggest that the landscape of energy-carrying molecules in ancient cells may not have mirrored that of their modern descendants. Evolutionary switches in the ATP or GTP specificities of enzymes are known to have occurred, for example in the early-evolved family of P-loop NTPases (8–10). Furthermore, experimental studies have demonstrated that such specificities can be quite mutable, mediated by even a single amino acid substitution (7, 11). Finally, though modern ATP- (or GTP-) binding enzymes can require high specificity for their cognate nucleotide (12, 13), many do exhibit promiscuous behavior and retain a lower level of activity with the alternate nucleotide (10, 14, 15). More broadly, substrate promiscuity has been observed in a number of reconstructed, phylogenetically inferred ancestral enzymes (16), though promiscuity as an inherent property of ancestral enzymes has been debated (17, 18). Regardless of the general trend, these findings together raise the possibility that ancestors of ATP-dependent enzymes may not have exhibited the same specificities. Thus, the requirement for ATP as life’s primary energy currency may not have been as stringent for ancient cellular organisms.

Here, we investigate this possibility by examining enzymatic ATP requirements in the evolutionary history of a critical microbial metabolism: biological nitrogen fixation. Life on Earth requires a sustained availability of fixed N, an essential element of many biological molecules. However, throughout Earth’s history, it is thought that surface N has existed primarily in the form of atmospheric dinitrogen (N_2_) (19), which is not directly usable for incorporation into biomolecules. To be biologically available, N_2_ must be fixed by reduction to ammonia (NH_3_) or oxidation to nitrogen oxides (NO_x_) (20–22). There is evidence from the geologic record for the emergence of nitrogenase enzymes as a means to reduce N_2_ to NH_3_ as early as 3.2 billion years ago (Ga) (23). Today, nitrogenase occurs in a wide array of microbes, from which only three isozymes are known: Mo-, V-, and Fe-nitrogenases, each distinguished by the composition of their active-site metal cofactor (24–26). For all three isozymes, two-component proteins (dinitrogenase and dinitrogenase reductase) work together to reduce N_2_ to NH_3_, utilizing electrons and MgATP derived from metabolism (25, 26). Mo-nitrogenase is the most commonly occurring and best studied (26–29) and the earliest to evolve (30).

The essentiality of MgATP in supporting N_2_ reduction by extant Mo-nitrogenase is well known. Intriguingly, divalent metal ions other than Mg^2+^ can support the ATP function [albeit with lower resulting N_2_ reduction rates (31)], yet ATP itself is absolutely required in the mechanism of N_2_ reduction by Mo-nitrogenase, with other nucleotide triphosphates (GTP, UTP, and CTP) not supporting N_2_ reduction (32). Based on these characteristics of extant nitrogenases, one might hypothesize that all ancient nitrogenases similarly had strict requirements for ATP. However, findings of generalist functions for other ancient enzymes (16) raise the possibility that ancestral nitrogenases may have had broader specificities. The specific sequence-level determinants of ATP dependence in nitrogenase are not well understood. Prior studies have demonstrated that ancestral nitrogenases have combinations of sequence features that are not represented by extant homologs (33), indicating the potential for epistatic interactions that might generate distinct, ancestral phenotypes. Importantly, the indispensability of ATP in ancient N_2_ fixation has not yet been experimentally verified, particularly given the early geochemical availability of other cofactors. It is thus currently unknown whether other nucleoside triphosphates, perhaps coupled to metal ions other than Mg^2+^, may have been capable of supporting an ancient Mo-nitrogenase early in its evolutionary history.

To address this longstanding question, we utilized an experimental paleogenetic approach to investigate the requirement of ATP for ancient biological nitrogen fixation. We reconstructed a Proterozoic ancestor of the nitrogenase component proteins, NifH_2_ and NifD_2_K_2_, followed by biochemical and biophysical characterization of the purified ancient proteins with a specific focus on ATP utilization. Our results provide direct laboratory evidence for the necessity of early enzyme cofactor usage of a key enzyme and underscore how biomolecular constraints can predominantly shape metal selection despite the far greater availability of metals with similar chemical properties in the environment.

## MATERIALS AND METHODS

### Ancestral sequence reconstruction

Nitrogenase phylogenetic construction and ancestral sequence reconstruction were performed as described by Garcia et al. (30, 34). Briefly, a nitrogenase protein sequence data set was curated by BLASTp (35) against the NCBI non-redundant protein database (accessed August 2020) using *Azotobacter vinelandii* NifH, NifD, and NifK query sequences. Sets of homologs for each nitrogenase subunit were aligned by MAFFT v7.450 (36) together with outgroup dark-operative protochlorophyllide oxidoreductase sequences (Bch/ChlLNB) and concatenated into a single alignment. Phylogenetic reconstruction by RAxML v8.2.10 was performed using the untrimmed, concatenated alignment and best-fit model parameters, LG + G + F, assessed by ModeFinder (37) implemented in IQ-TREE v.1.6.12 (38). Clade support was evaluated by the SH-like aLRT (39). Ancestral sequence reconstruction was performed by PAML v4.9j (40) using the same model parameters used for phylogenetic reconstruction, generating the ancestral nitrogenase NifHDK protein sequence “Anc^AK029^” targeted in the current study (see Fig. 2A). Nitrogenase phylogenies were visualized using the ggtree v.3.8.0 R package.

### *A. vinelandii* strain engineering

Strains and plasmids used in the present study are listed in Table S1. The most probable ancestral NifHDK protein sequence (i.e., the sequence with the most probable ancestral residue per site) reconstructed for node Anc^AK029^ was reverse-translated and codon-optimized for expression in *A. vinelandii* (wild type [WT]), as described by Garcia et al. (34). Sites for which the Anc^AK029^ and WT amino acid residues were identical were assigned the WT codon, and sites for which the ancestral and WT amino acid residues differed were assigned a random codon weighted by *A. vinelandii* codon frequencies (Codon Usage Database, https://www.kazusa.or.jp/codon/). The optimized Anc^AK029^ *nifH*, *nifD*, and *nifK* nucleotide sequences were concatenated together with *A. vinelandii* intergenic regions and 1,000-bp flanking sequences to direct homologous recombination at the native *nifHDK* locus in the *A. vinelandii* genome. In addition, an “ASWSHPQFEK” Strep-II tag was appended to the N-terminus of ancestral NifD for downstream affinity purification. The concatenated sequence was synthesized and cloned into pUC19 (GenScript, Piscataway, NJ, USA), yielding plasmid pAnc^AK029^.

Genomic integration of ancestral *nifHDK* into *A. vinelandii* followed Garcia et al. (34), using established methods for transformation and homologous recombination (41), Genetically competent cells were prepared from the non-diazotrophic *A. vinelandii* parent strain, ∆nif, containing disruptions to all three *nif*, *vnf*, and *anf* nitrogenase gene clusters (42) by growth in Mo- and Fe-deficient BN medium. The Anc^AK029^ strain harbors a clean replacement of *nifHDK* with a kanamycin resistance cassette (KanR). Competent cells were transformed by congression (i.e., the coincident introduction of two unrelated genetic modifications) (41) using ~1 µg of plasmid pDB303 and 1 µg of digested (ScaI-HF, New England BioLabs) plasmid pAnc^AK029^, the former containing the rifampicin resistance determinant *rpoB113* (RifR). Transformants were screened for rifampicin resistance and loss of kanamycin resistance and confirmed via both Sanger sequencing (primers listed in Table S1) and Oxford Nanopore whole-genome sequencing (Plasmidsaurus, Eugene, OR, USA). Transformants were stored at −80°C in phosphate buffer with 7% DMSO.

### *A. vinelandii* growth

Cultures were grown in liquid or solid Burk’s media (BN medium) with 1 µM Na_2_MoO_4_ and 10 mM ammonium acetate. To induce diazotrophic growth, Burk’s media without ammonium acetate was also used (B medium). Fifty milliliter seed cultures in BN medium were grown for 24 hours at 30°C with a 300-rpm double orbital agitation for nitrogenase expression and growth rate quantification.

Growth rate analysis followed the protocol described in Carruthers et al. (43), as summarized below. Three biological replicates of Anc^AK029^ and WT strains were inoculated from the 24-hour seed cultures into liquid B medium at an optical density at 600 nm (OD_600_) of 0.05. The cultures were aliquoted into a 96-well, flat-bottom plate (Greiner Bio-One) and sealed with a Breathe-Easy adhesive membrane (Diversified Biotech). The plate was placed in a SPECTROstar Nano Microplate Reader (BMG Labtech, Ortenberg, Germany) and incubated at 30°C with a 300-rpm double orbital agitation. The OD_600_ of the cultures was measured every 30 minutes for 90 hours. The R package Growthcurver was used to calculate doubling time and visualize data (44). Statistical significance was determined using one-way ANOVA and *post hoc* Tukey HSD test.

### Protein quantification

Protein quantification of Strep-II-tagged NifD was conducted for Anc^AK029^ and DJ2102 (containing a Strep-II-tagged WT NifD) strains following the protocol in Garcia et al. (34) with modifications as described below. Flasks containing 100 mL B medium were inoculated to an OD_600_ of 0.01 and grown diazotrophically. Cell pellets were harvested after 4 hours and stored at −80°C. TE lysis buffer (10 mM Tris, 1 mM EDTA, and 1 mg/mL lysozyme) was prepared and used to resuspend the cell pellets. The resuspended cell pellets were vortexed lightly and heated for 10 minutes at 95°C. The lysates were centrifuged for 15 minutes at 5,000 rpm. The supernatants were analyzed for total protein quantification using the Pierce BCA Protein Assay kit (ThermoFisher). The lysates were normalized to 200 µg total protein and diluted 1:1 with 2× Laemmli buffer before performing polyacrylamide gel electrophoresis (PAGE). The proteins from the PAGE gel were transferred to a nitrocellulose membrane (ThermoFisher). The membrane was stained with Revert 700 Total Protein Stain (LI-COR) and imaged with an Odyssey Fc Imager (LI-COR). The membrane was destained using Revert Destaining Solution (LI-COR) and blocked for 1 hour using 5% non-fat milk dissolved in PBS (137 mM NaCl, 2.7 mM KCl, 10 mM Na_2_HPO_4_, and 1.8 mM KH_2_PO_4_). The membrane was rinsed with PBS and 0.01% Tween-20 mixture (PBS-T) to remove residual blocking solution. The membrane was then incubated with primary Strep-II antibody (Strep-MAB-Classic, IBA Lifesciences, 1:5,000 in 0.2% BSA) and gently rocked at 10°C for 16 hours and then at room temperature for two additional hours. The membrane was rinsed with PBS-T and incubated with 1:15,000 IRDye 680RD goat anti-mouse in LI-COR blocking buffer for 2 hours at room temperature. The membrane was then imaged again using an Odyssey Fc Imager (LI-COR).

### Purification reagents and procedures

Reagents were obtained from Sigma-Aldrich (St. Louis, MO, USA) or Fisher Scientific (Fair Lawn, NJ, USA) and used without further purifications. Argon and dinitrogen gases were purchased from Air Liquide America Specialty Gases (Plumsteadville, PA, USA). All manipulations of proteins and buffers were done anaerobically in septum-sealed serum vials and flasks utilizing a vacuum Schlenk line under argon or dinitrogen atmospheres and gas-tight syringes.

### Protein purification and quantification

NifD_2_K_2_ and NifH_2_ proteins were expressed and purified from *Azotobacter vinelandii* strains DJ2102 (Extant Strep-NifD_2_K_2_), DJ884 (Extant NifH_2_), and Anc^AK029^ (Ancient Strep-NifD_2_K_2_ and NifH_2_) by previously published methods (45, 46). Protein concentration was quantified by the biuret method (47) with bovine serum albumin as a standard, and purity was assessed at ≥90% by SDS-PAGE with Coomassie blue staining. The H^+^ reduction activity of extant NifD_2_K_2_ was assessed at 8.5 nmol H_2_/nmol NifD_2_K_2_/s, which is within the established range for fully active NifD_2_K_2_ from *Azotobacter vinelandii* (48–51). The H^+^ reduction activity of Anc^AK029^ NifD_2_K_2_ was assessed at 3.8 nmol H_2_/nmol NifD_2_K_2_/s.

### Electron paramagnetic resonance

Electron paramagnetic resonance (EPR) samples of extant and Anc NifD_2_K_2_ and NifH_2_ were prepared with 50 µM of each protein. NifH_2_ was prepared in a buffer of 50 mM Tris (pH 7.5), 100 mM NaCl, and 10 mM dithionite. NifD_2_K_2_ was prepared in a buffer of 100 mM MOPS (pH 7), 5 mM ATP, 10 mM phosphocreatine, 7.5 mM MgCl_2_, 0.2 mg/mL creatine phosphokinase, and 1.3 mg/mL BSA. Prepared samples were frozen in a pentane/liquid N_2_ slurry. Continuous-wave X-band EPR spectra were recorded using a Bruker ESP-300 spectrometer with an EMX PremiumX microwave bridge and an EMX^PLUS^ standard resonator in perpendicular mode, equipped with an Oxford Instruments ESR900 continuous helium flow cryostat using VC40 flow controller for helium gas. Spectra were recorded at the following conditions: temperature, ~12 K; microwave frequency, ~9.38 GHz; microwave power, 20 mW; modulation frequency, 100 kHz; modulation amplitude, 8.14 G; and time constant, 20.48 ms. Each spectrum is the sum of five scans.

### Substrate reduction assays

Assays were performed in 9.4 mL vials with a nucleotide regeneration buffer (6.7 mM MgCl_2_, 30 mM phosphocreatine, 5 mM ATP, 0.2 mg/mL creatine phosphokinase, and 1.2 mg/mL BSA) and the reductant 10 mM sodium dithionite in 100 mM MOPS buffer at pH 7.0. Reaction vials were made anaerobic and put under an atmosphere of N_2_ gas. In assays that tested different nucleoside triphosphates (GTP, ITP, and UTP), the ATP in the buffer was replaced with the appropriate nucleoside triphosphate at the same concentration. Controls with no nucleoside triphosphate added had no activity. In assays that tested alternative divalent metal ions (Mn^2+^, Fe^2+^, and Co^2+^), the Mg^2+^ was replaced with the appropriate metal at the same concentration. Controls with no metal added had no activity. All assays were performed with 0.42 µM NifD_2_K_2_ and 8.4 µM NifH_2_. NH_3_ was quantified using a fluorescence protocol (52) with some modifications. An aliquot of the sample was added to a solution containing 200 mM potassium phosphate pH 7.3, 20 mM o-phthalaldehyde, and 3.5 mM 2-mercaptoethanol and incubated for 30 minutes in the dark. Fluorescence was measured at λ_excitation_ of 410 nm and λ_emission_ of 472 nm, and NH_3_ was quantified using a standard generated with NH_4_Cl. H_2_ was measured and quantified using a molecular sieve 5A column and thermal conductivity detector.

### ATP hydrolysis

Assays were performed in a reaction buffer with 10 mM MgATP and 10 mM dithionite in 100 mM MOPS buffer at pH 7.3, without a MgATP regeneration system. The protein concentrations were 0.42 µM NifD_2_K_2_ and 8.4 µM NifH_2_. The ratios of hydrolyzed ATP per electron transferred for product formation under N_2_ were determined by quantification of the total amount of P_i_ versus total amount of electrons in the products H_2_ and NH_3_ as previously described (53).

## RESULTS AND DISCUSSION

**Fig 1:**
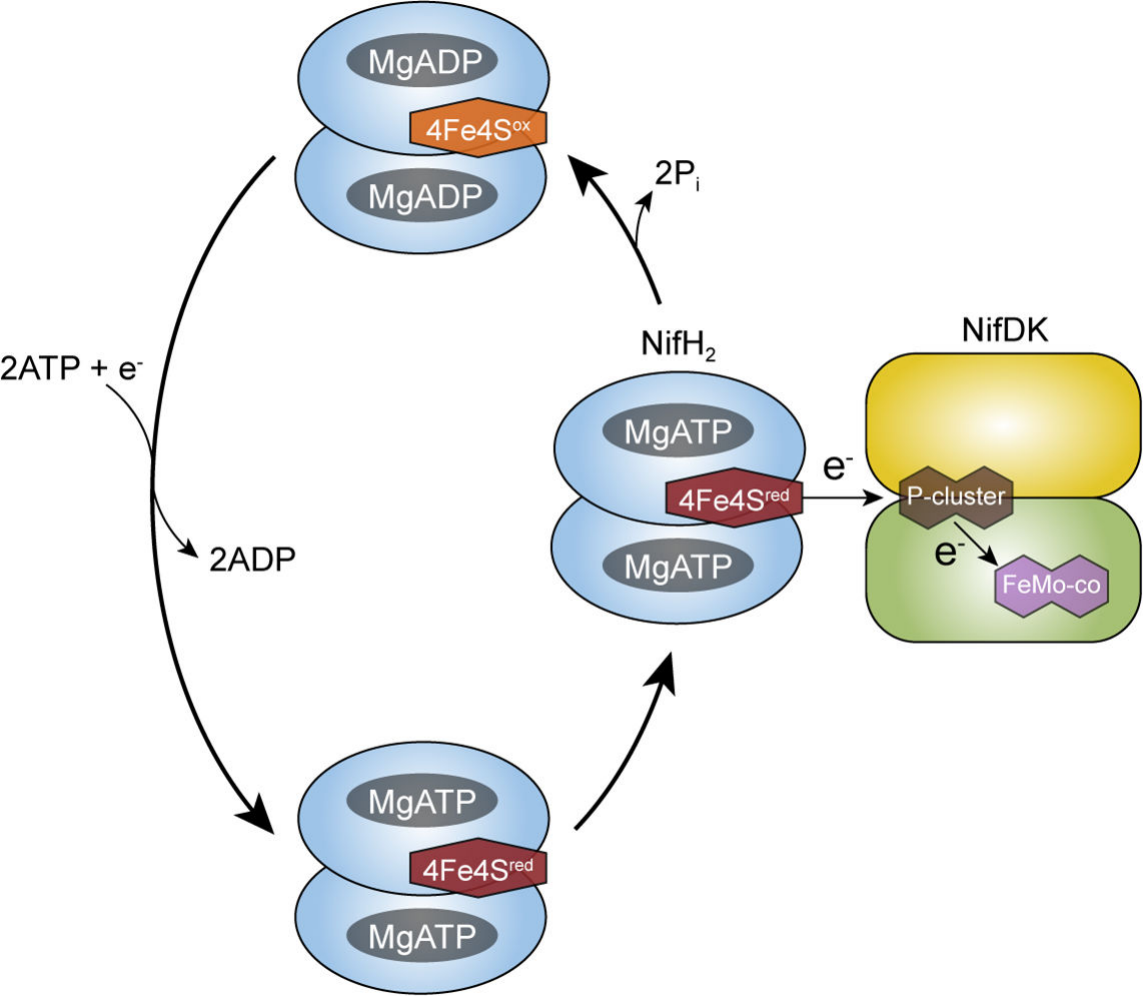
NifH_2_ cycle of Mo-nitrogenase. The NifH_2_ homodimer is shown in blue; it houses two nucleotide-binding sites and a [4Fe-4S] redox active cluster. One catalytic half (NifDK) of the NifD_2_K_2_ heterotetramer is shown in yellow (K) and green (D); each catalytic half houses one [8Fe-7S] (P-cluster) and a [7Fe-9S-Mo-C-homocitrate] (FeMo-co) active site metallocluster. When NifH_2_ binds to NifDK, an electron is transferred from the [4Fe-4S] cluster to FeMo-co mediated by P-cluster. NifH_2_ then hydrolyzes two ATP, dissociates with bound MgADP and an oxidized [4Fe-4S] cluster. MgADP is then exchanged for MgATP and the [4Fe-4S] cluster is reduced, preparing NifH_2_ for binding and electron transfer.


N_2_ reduction by extant Mo-nitrogenase involves the sequential delivery of electrons from the dinitrogenase reductase (NifH_2_) component to the dinitrogenase (NifD_2_K_2_) component, the latter containing the active-site metal cofactor where N_2_ is reduced (Fig. 1) (26, 54). Each electron delivery event from NifH_2_ to NifD_2_K_2_ requires the hydrolysis of a minimum of two MgATP molecules to two MgADP and two P_i_. Under optimal conditions, the minimal reaction stoichiometry can be written as shown in equation 1 (29, 53, 54).

(1) $$N_{2}+8H^{+}+16MgATP+8e^{-}\to 2NH_{3}+H_{2}+16MgADP+16P_{i}$$

Within this reaction, ATP serves several functions. ATP binds to NifH_2_, inducing conformational changes in the protein that impact its [4Fe-4S] cluster and affinity for binding to NifD_2_K_2_ (27, 54, 55). Furthermore, there is growing evidence that the reductase protein induces conformational changes within NifD_2_K_2_ that contribute to substrate binding and reduction (54, 56, 57). Finally, the hydrolysis of ATP and release of the two P_i_ molecules signal the release of NifH_2_ with two bound MgADP from NifD_2_K_2_, with the energy from ATP hydrolysis being used to dissociate the two-component proteins (50, 53, 58, 59). The released NifH_2_ is reduced by cellular electron carrier proteins (e.g., ferredoxin or flavodoxin) or non-cellular reductants, such as dithionite (27). The two bound MgADP molecules are exchanged with two MgATP molecules, readying NifH_2_ for another round of electron transfer to NifD_2_K_2_ (Fig. 1) (27, 50, 58).

**Fig 2:**
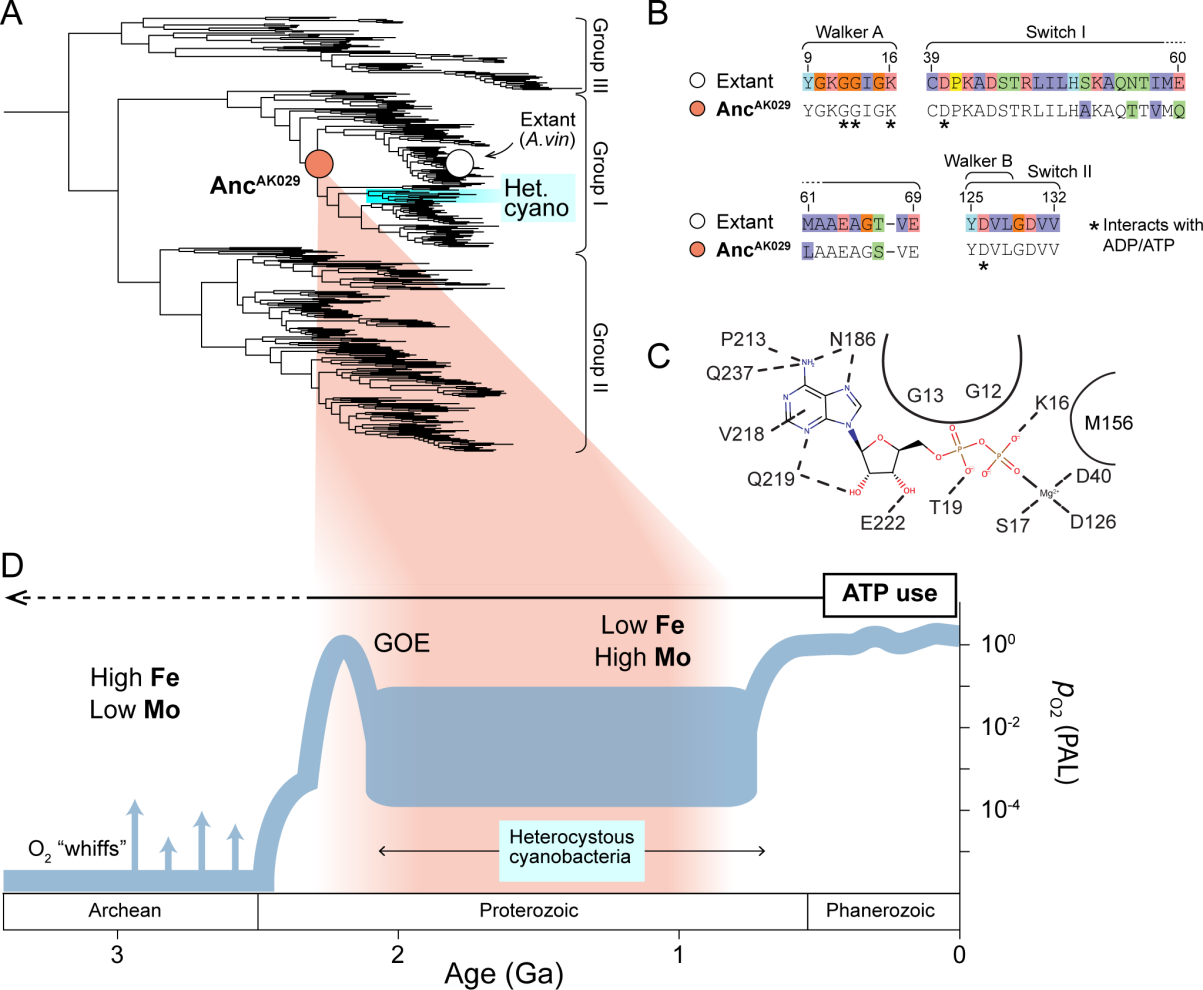
Evolution of the ATP/ADP-binding site in nitrogenase NifH. (**A**) Nitrogenase NifHDK protein phylogeny. Phylogenetic positions of ancestral (Anc^AK029^) and extant *A. vinelandii* (“Extant *A. vin”*) nitrogenase variants discussed in the main text are highlighted by colored circles. (**B**) Protein sequence alignment of NifH ATPase signature motifs (labeled) (54) site index from *A. vinelandii* NifH. (**C**) Residue-level intermolecular interactions in the ADP-binding site of *A. vinelandii* NifH (PDB 1FP6). All displayed residues are conserved between Anc^AK029^ and WT. Curved lines delineate residues with nonspecific interactions that shape the binding site. (**D**) Estimated age range of nitrogenase ancestors mapped to Earth’s environmental history. Nitrogenases hosted by heterocystous cyanobacteria provide a minimum age constraint based on oldest fossil evidence (60). Atmospheric oxygenation plot and relative marine metal abundances are from Lyons et al. (61).

We began our study by identifying an ancestral nitrogenase protein (Anc^AK029^) to experimentally test ATP utilization in early biological nitrogen fixation (Fig. 2A). We drew from a prior data set of ancestral nitrogenase proteins, inferred from a maximum likelihood phylogenetic tree of concatenated NifHDK protein sequences representative of known nitrogenase diversity (34). Specifically, we selected the Anc^AK029^ ancestor based on two criteria: the ancestral protein must (i) occupy a deep position in the nitrogenase phylogeny while having a reasonably well-constrained age and (ii) belong to the direct evolutionary lineage of our laboratory model bacterium *Azotobacter vinelandii* (*A. vinelandii*) (Fig. 2A). Regarding the first criterion, we infer that Anc^AK029^ has a Proterozoic age range of ~0.7–2.4 Ga. The minimum age of ~0.7 Ga is constrained by more recently diverged, heterocystous cyanobacterial nitrogenases (60). The maximum age is constrained by the position of the Anc^AK029^ node within the “Group I” nitrogenase clade, which is primarily hosted by aerobic and facultatively anaerobic bacteria (34). Therefore, Anc^AK029^ likely existed after the early oxygenation of the Earth’s surface environment (Great Oxidation Event) at ~2.4 Ga and subsequent diversification of oxygen-tolerant microbes (61).

To gain initial insight into the nucleotide specificity of Anc^AK029^ nitrogenase, we analyzed its global sequence similarity to the Nif nitrogenase of *A. vinelandii* (hereafter simply referred to as the extant nitrogenase), as well as its ancestral features within the inferred nucleotide-binding pocket. Anc^AK029^ NifH, NifD, and NifK proteins together have ~72% amino acid sequence identity to those of extant nitrogenase. Sequence-level identity is highest between ancestral and extant NifH proteins (~87%) and lowest between NifK proteins (~61%). Known ATPase sequence motifs are well conserved in NifH, including the nucleotide-binding P-loop, Walker B motif, and “switch I and II” motifs (Fig. 2B) (8, 62). The immediate nucleotide-binding site within NifH includes 14 residues, all of which are identical between Anc^AK029^ and extant nitrogenase (Fig. 2C). Of these 14 residues, 10 are also universally conserved across all extant nitrogenases in our data set. Nevertheless, given the lack of knowledge on the sequence-level determinants of ATP specificity in nitrogenase, as well as the potential impact of even single amino acid substitutions on ATP specificity (7, 11), we proceeded to experimentally investigate the nucleotide dependence of Anc^AK029^.

### Bacterial growth and biochemical characterization

Expression of ancient, reconstructed enzymes in extant organisms has inherent challenges (63). A particular complexity of nitrogenase is the dependence on interactions with multiple protein partners in order to mature a functional enzyme. Thus, a first assessment of the functionality of the inferred ancient proteins is that they can be matured and support diazotrophic growth in an extant organism.

**Fig 3:**
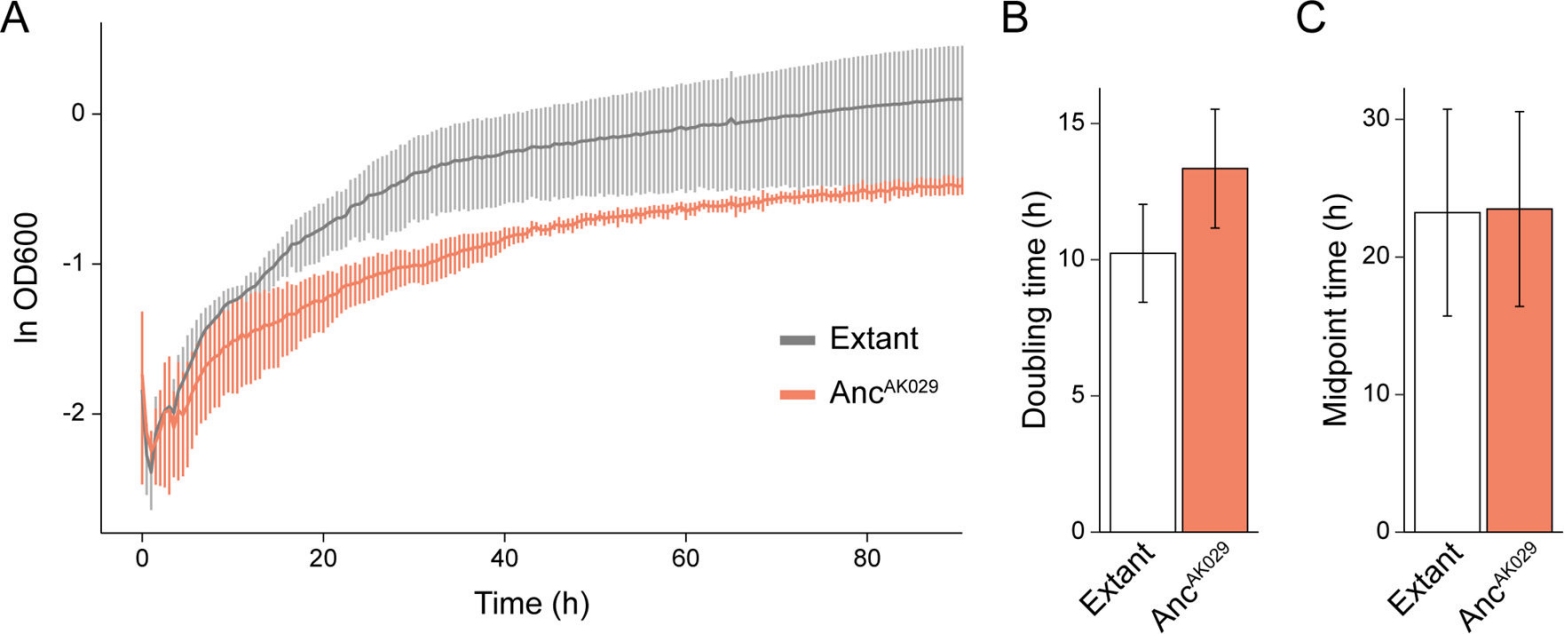
Diazotrophic growth of WT and Anc^AK029^ *A. vinelandii* strains. (**A**) Diazotrophic growth curve of *A. vinelandii* expressing either the WT or Anc^AK029^ nitrogenase proteins. Line plots represent the average ln OD_600_ of three biological replicates per strain, and error bars indicate ±1 SD. (**B**) Doubling times of WT and Anc^AK029^. (**C**) Midpoint times of WT and Anc^AK029^. (**B and C**) Bars represent the average of three biological replicates per strain, and error bars indicate ±1 SD.

Synthetic *nifHDK g*enes encoding the Anc^AK029^ nitrogenase were genomically integrated into the modern nitrogen-fixing model bacterium, *Azotobacter vinelandii*, replacing its native Mo-nitrogenase genes. The results of growth experiments for Anc^AK029^ relative to the extant strain under nitrogen-fixing conditions are shown in Fig. 3. Growth of the Anc^AK029^ strain is slightly slower overall, but within the error of the extant control, indicating that the ancient nitrogenase is being expressed and is sufficiently functional for N_2_ reduction to support cell growth. Expression was further verified by Western blot (Fig. S1), which showed a strong overexpression of the nitrogenase proteins in Anc^AK029^ relative to the extant strain.

Protein purification of Strep-II-NifD_2_K_2_ by Strep-Tactin affinity chromatography (45) and NifH_2_ by anion exchange and size exclusion chromatography (46) yielded fractions of high purity as determined by SDS-PAGE (Fig. S2). Densitometry analysis of the SDS-PAGE bands revealed a D:K subunit stoichiometry of 1.44 for Anc^AK029^ versus 0.93 for extant. Fully occupied NifH_2_ contains a [4Fe-4S] cluster with 4 nmol of Fe/nmol NifH_2_. NifD_2_K_2_ contains two FeMo-cofactors (7Fe-9S-Mo-C-homocitrate) and two P-clusters (8Fe-7S) and thus has 2 nmol Mo and 30 nmol Fe/nmol NifDK (Fe:Mo = 15) (24). Metal analysis by ICP-MS showed Anc^AK029^ NifH_2_ at 2.4 nmol Fe/nmol NifH_2_ (~60% occupied) and Anc^AK029^ NifD_2_K_2_ at 0.8 nmol Mo/nmol NifD_2_K_2_ and 12.1 nmol Fe/nmol NifD_2_K_2_ (~40% occupied and Fe:Mo = 14.7). EPR analysis of the [4Fe-4S] cluster of NifH_2_ and FeMo-co of NifD_2_K_2_ in Anc^AK029^ revealed spectra like those established in extant proteins, with a slight shift of the FeMo-co *g*-value (Fig. S3). Since the second sphere coordination environment can tune the electronic property of FeMo-co, these shifts in *g*-values are common when comparing the spectra of FeMo-co from other organisms or even single amino acid substitutions in extant NifD_2_K_2_ (64, 65). Substrate reduction assays showed activities for Anc^AK029^ that are ~40% of extant MoFe protein for N_2_ reduction under 1 atm N_2_. This decreased activity correlates closely with the metal occupancy, and in combination with the densitometry analysis, it reveals some combination of incomplete maturation or metal loading in the cell. The pure protein is stable over both freeze-thaw cycles and when thawed on the bench over the course of multiple assays.

### Alternative nucleotides and divalent metals

**Fig 4:**
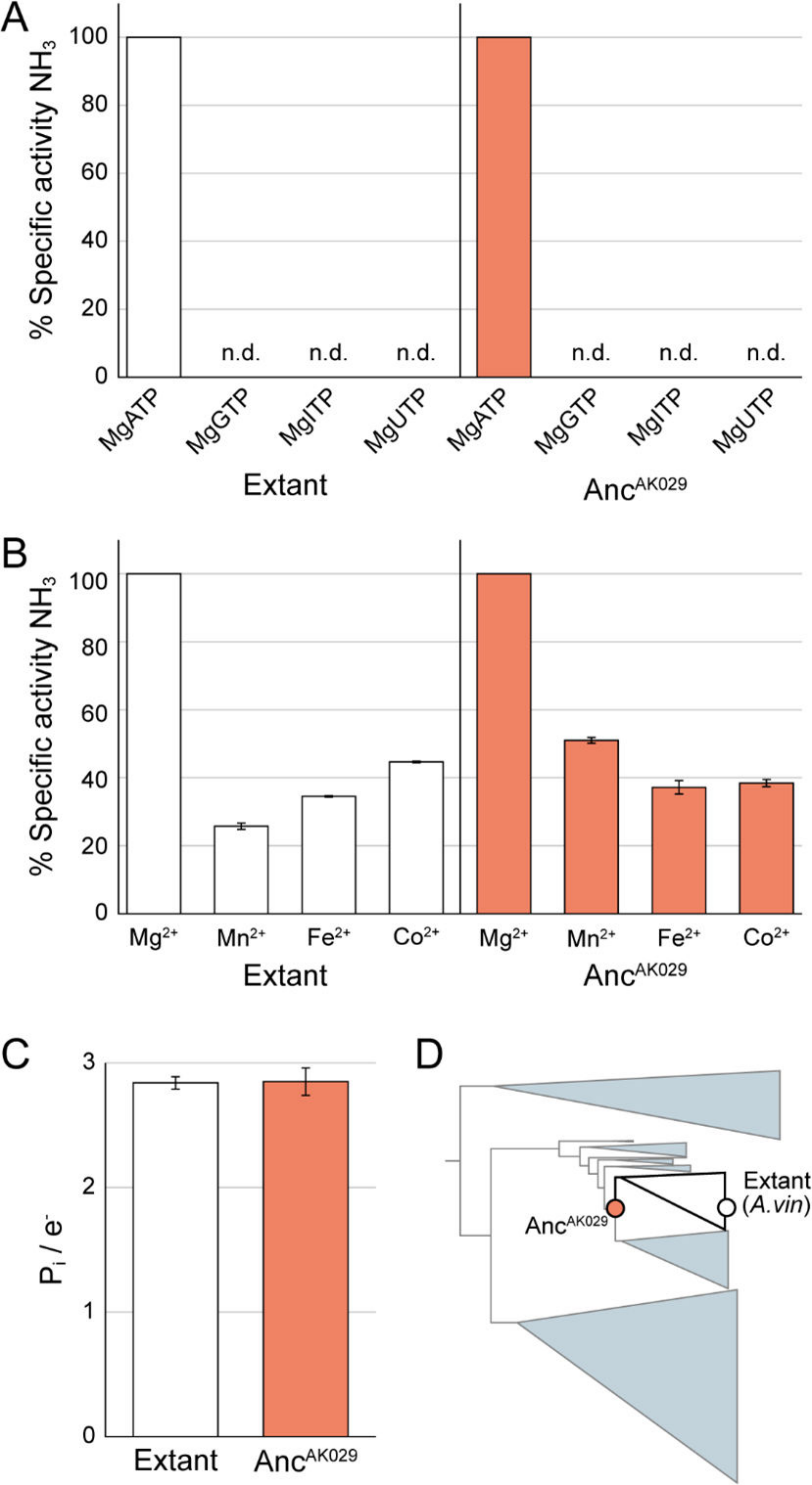
Ancestral nitrogenase behavior with alternative nucleotides and divalent metals. (**A**) Specific activity for N_2_ reduction by extant and Anc^AK029^ NifHDK with different nucleotides complexed with Mg^2+^. Shown are percent specific activities for NH_3_ formation relative to the maximum activity seen with MgATP. n.d., not detectable (specific activity of Anc^AK029^ is ~40% of extant; specific activities are listed in Table S1). (**B**) Specific activity for N_2_ reduction by extant and Anc^AK029^ NifHDK with ATP and different divalent metal ions. Shown are percent specific activities for NH_3_ formation relative to the maximum activity seen with Mg^2+^ (specific activity of Anc^AK029^ is ~40% of extant; specific activities are listed in Table S1). Bars represent the average of three replicates, and error bars represent ±1 standard deviation. (**C**) Inorganic phosphate released per electron transferred during N_2_ reduction. Shown is inorganic phosphate measured normalized to total electrons in products H_2_ and NH_3_ for extant and Anc^AK029^ nitrogenases when allowed to turn over for 2 min under 1 atm of N_2_. Bars represent the average of three replicates, and error bars represent ±1 standard deviation. (**D**) Simplified nitrogenase phylogeny shown in Fig. 2A indicates evolutionary relationship between variants analyzed in panels **A–C**.

The nucleoside triphosphates (e.g., ATP, GTP, ITP, UTP, and CTP) used by life are each structurally unique and available in the Proterozoic environment (1), and they all yield a similar amount of energy upon hydrolysis of the gamma phosphate. Previous studies have shown that extant NifH_2_ can hydrolyze different nucleotides when bound to NifD_2_K_2_, but only ATP supports electron transfer and substrate reduction at NifD_2_K_2_ (32). These results support the hypothesis that ATP is not simply providing energy for nitrogenase but that there is something unique to its structure that promotes electron transfer and substrate reduction (54). Similar results were found in N_2_ reduction assays here with extant and Anc^AK029^ nitrogenases. No substrate reduction activity or nucleotide hydrolysis was observed for the ancient enzyme with MgGTP, MgITP, and MgUTP (Fig. 4A). These findings provide evidence for a specific requirement for ATP in the evolution of nitrogenase.

Hydrolysis of ATP by enzymes usually requires a divalent metal ion, facilitating interactions with the enzyme and stabilizing the hydrolysis transition state. The availability of various divalent metal ions has changed throughout Earth’s history, particularly with the oxygenation of the atmosphere (66). While many divalent metal ions can serve in the hydrolysis of ATP, Mg^2+^ is the most widely used in extant biological processes and typically supports the highest efficiency and rates (31, 67). Mg^2+^ is also widely used in many proposed Archaean-aged metabolisms (68) and has likely remained abundant in the environment (69), suggesting that Mg^2+^ would have been available for use by early nitrogen-fixing bacteria.

N_2_ reduction assays were performed with different divalent metal ions and ATP for both the extant and Anc^AK029^ nitrogenases (Fig. 4B). While Mg^2+^ supported the highest activity in both extant and Anc^AK029^, it is not a strict requirement for either enzyme. For example, Mn^2+^, Fe^2+^, and Co^2+^ each showed between 30% and 50% of the rates of substrate reduction in the Anc^AK029^ nitrogenase compared to the rates with Mg^2+^ (Fig. 4B). These findings reveal that while Mg^2+^ provides the highest specific activities for N_2_ reduction for both extant and the Anc^AK029^ enzymes, other divalent metals can also support activity and could be utilized depending on the metals available in the environment.

### ATP/e^−^ usage

Extant NifH_2_ hydrolyzes approximately two ATP only while complexed with NifDK, which is coupled to the transfer of a single electron per association event (Fig. 1) (50, 53, 54). This ratio of ATP/e^−^ is a marker of the specificity and control of the system. While NifH_2_ has all the makings of a canonical ATPase (54, 70), only the full NifH_2_-NifDK complex shows any detectable ATPase activity. This phenomenon allows for the efficient use of costly ATP and reducing equivalents and has been suggested to involve mechanisms that promote N_2_ binding and reduction (56, 57), To test the linkage of MgATP hydrolysis to electrons transferred to substrates, the inorganic phosphate released during N_2_ reduction assays was measured to quantify the ATP hydrolyzed. The amount of ATP hydrolyzed was compared to the total electrons transferred by totaling all electrons in the products H_2_ and NH_3_. As can be seen in Fig. 4C, Anc^AK029^ nitrogenase shows a P_i_/e^−^ ratio identical to that measured for extant nitrogenase of ~2.8 ATP/e^−^, which is in agreement with prior studies on extant nitrogenase (53). These results are further evidence for the tight linkage between MgATP hydrolysis and substrate reduction for both Anc^AK029^ and extant nitrogenases.

### Conclusion

Energy transfer to drive enzymatic reactions is a fundamental feature of life. However, understanding the evolution of the cellular suite of energy-carrying molecules has been stalled by the lack of investigative strategies to directly test the nucleotide triphosphate specificities of key ancient metabolic enzymes. Paleogenetic approaches like those employed in the present work enable functional insights that are not possible by examining extant enzymes alone. Ancestral sequence reconstruction has previously been used to investigate the evolutionary drivers of structural complexity (71), the origins of novel enzymatic activity and specificity (72), and ancient biosignature generation by geobiologically critical enzymes (73). Recently, ancestral nitrogenase enzymes were resurrected, providing evidence of long-term conservation of the N_2_-binding mechanism in biological nitrogen fixation (34). Now, our finding that the strict requirement for ATP by nitrogenase extends back to the Proterozoic not only addresses this gap by providing direct evidence of ancient biological ATP usage but also underscores its essential role for a microbial metabolism that has limited the biosphere billions of years (66, 74).

Extant Mo-nitrogenase demonstrates a unique relationship with ATP. The system not only requires the energy from the hydrolysis of ATP but also the binding of ATP itself is a strict mechanistic requirement for electron transfer from NifH_2_ to NifD_2_K_2_ and the ability to bind and reduce N_2_. Furthermore, while NifH_2_ has all the canonical sequence motifs of an ATPase, it is only the NifH_2_-NifD_2_K_2_ complex that hydrolyzes two ATP coupled to the transfer of a single electron. In this way, the system exerts tight control over the use of valuable ATP and reducing equivalents. Here, it is demonstrated that these specific aspects of the ATP-dependent mechanism of nitrogenase likely emerged early in the evolution of this enzyme. The reconstructed Anc^AK029^ shows strong conservation of ATP-relevant sequence motifs in NifH_2_, supports diazotrophic growth in *A. vinelandii*, and the purified enzyme is stable and active for N_2_ reduction. Like extant nitrogenase, N_2_ reduction activity in Anc^AK029^ is seen exclusively with ATP; alternative nucleotides (GTP, UTP, and ITP) are neither hydrolyzed nor support catalysis in the system. ATP hydrolyzed per electron transferred in Anc^AK029^ is identical to extant nitrogenase at ~2.8 ATP/e^−^. Finally, a number of divalent metals (Mn^2+^, Fe^2+^, and Co^2+^) are shown to support the function of ATP, but none approach the rates supported by Mg^2+^.

What is notable is the specificity of MgATP to the Mo-nitrogenase mechanism and, as evidenced here, how early this relationship formed, particularly given that the activities of other ATPases can be supported by other nucleoside triphosphates (10). The complexity, specificity, and control of the Mo-nitrogenase mechanism are testaments to the challenge of N_2_ reduction and the early evolution of these features speaks to the demand for a suitable catalyst by early life and the unique role that ATP played in meeting that demand.

## Data Availability

Additional data are available in the supplemental material. All unique biological materials (i.e., *Azotobacter* strains) will be available upon request. Other protein accession IDs are as follows: extant nitrogenase molybdenum-iron protein alpha chain—NifD, UniProtKB P07328; extant nitrogenase molybdenum-iron protein beta chain—NifK, UniProtKB P07329; and extant nitrogenase iron protein 1—NifH, UniProtKB P00459.

## SUPPLEMENTAL MATERIAL

Supplemental material mbio.01271-24-s0001.pdf Supplemental figures and tables.

ASM does not own the copyrights to Supplemental Material that may be linked to, or accessed through, an article. The authors have granted ASM a non-exclusive, world-wide license to publish the Supplemental Material files. Please contact the corresponding author directly for reuse.
