# Supplementary material for: Ancient nitrogenases are ATP dependent
Source: mBio. 2024 Jun 13;15(7):e01271-24. doi: 10.1128/mbio.01271-24 (PMC11253609; doi:10.1128/mbio.01271-24)
Supplement: Supplemental material — Supplemental figures and tables. [file mbio.01271-24-s0001.pdf]

Ancient Nitrogenases are ATP Dependent

Derek F. Harris[a], Holly R. Rucker[b], Amanda K. Garcia[b], Zhi-Yong Yang[a], Scott D. Chang[b], Hannah Feinsilber[a], Betül Kaçar[b#], Lance C. Seefeldt[a#]

[a] Department of Chemistry and Biochemistry, Utah State University, Logan, UT 84322

[b] Department of Bacteriology, University of Wisconsin–Madison, Madison, WI 53706

Running Title: Origins of ATP in Nitrogen Fixation.

Derek F. Harris and Holly R. Rucker contributed equally. Author order was determined on the basis of seniority.

[#]Corresponding authors: Lance Seefeldt, email: lance.seefeldt@usu.edu; Ph. +1.435.797.3964. Betül Kaçar, betul.kacar@wisc.edu; Ph. +1.608.262.2914.

Supplemental figures

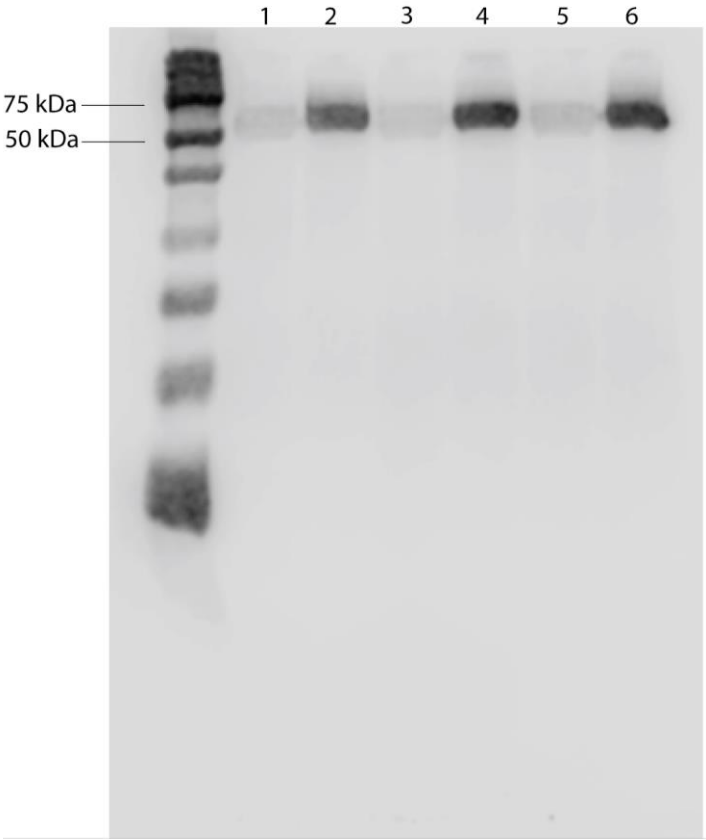

Figure S1. Immunodetection of Strep-II-tagged NifD in WT (lanes 1, 3, and 5) and Anc *A. vinelandii* strains (lanes 2, 4, and 6). Detection of Strep-II-tagged NifD was determined using an anti-Strep antibody. Each lane represents one biological replicate (three replicates per strain). The expected size of NifD is 55.8 kDa.

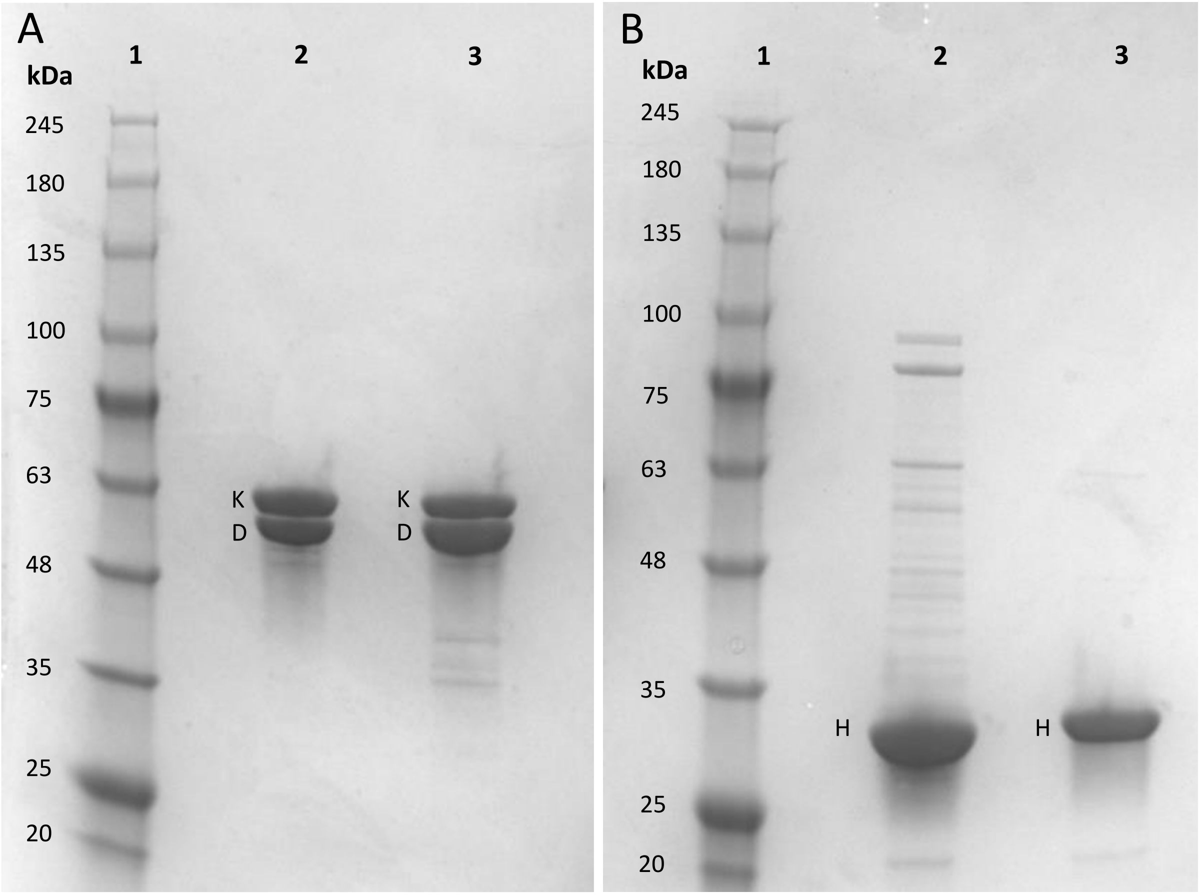

C

	Intensity	Molecular Weight (kDa)	Intensity/kDa	Stoichiometry (D:K)
Extant K	19,785,513	57,530	343.92	0.93
Extant D	17,344,927	54,230	319.84	
Anc K	18,160,805	56,100	323.72	1.44
Anc D	24,734,047	53,020	466.50	

Figure S2. Coomassie stained SDS-PAGE and densitometry analysis. A) Shown is protein ladder (lane 1) and purified Extant (lane 2) and Anc (lane 3) NifD and NifK subunits. B) Shown is protein ladder (lane 1) and purified Extant (lane 2) and Anc (lane 3) NifH. C) Densitometry analysis of NifDK.

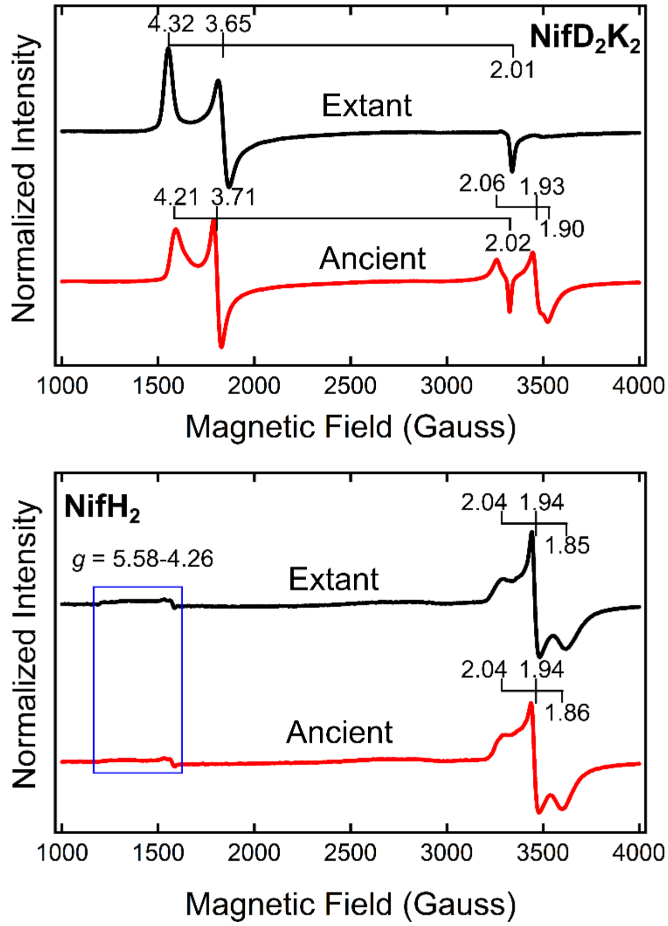

Figure S3. Continuous-wave (CW) EPR spectra of extant (black traces) and ancient (red traces) $NifD_2K_2$ (top panel) and $NifH_2$ (bottom panel) proteins in the dithionite-reduced resting state. The EPR spectra were recorded from samples containing 50 μM of each protein at 12 K and 20 mW microwave power. Each spectrum is a sum of 5 scans. The ancient $NifD_2K_2$ displayed a similar S = 3/2 spin signal as that in the extant $NifD_2K_2$ with slight difference in rhombicity as indicated by the g values (top panel). Moreover, an S = 1/2 signal in the $g \sim 2$ region (g = 2.06, 1.93, and 1.90) might originate from an immature P-cluster species (1). The ancient $NifH_2$ exhibited almost identical EPR features as those of the extant $NifH_2$, with the blue box highlighting the low intensity high spin states (bottom panel).

Table S1. Specific activities for Figure 4.

	N_2 reduction specific activity (nmol NH_3/nmol $NifD_2K_2$/s)							
	Different metal ions with ATP				Different nucleotides with Mg^{2+}			
**Nitrogenase**	**Mg^{2+}**	**Mn^{2+}**	**Fe^{2+}**	**Co^{2+}**	**ATP**	**GTP**	**UTP**	**ITP**
**Extant**	2.24 ± 0.13	0.68 +/- 0.03	0.91 ± 0.01	1.17 ± 0.01	2.24 ± 0.13	n.d.	n.d.	n.d.
**Anc^{AK029}**	0.86 ± 0.07	0.44 ± 0.01	0.32 ± 0.02	0.33 ± 0.01	0.86 ± 0.07	n.d.	n.d.	n.d.

Table S2. Strains, plasmids, and primers used in the current study.

Type	Designation	Source	Additional Information
A. vinelandii strain	WT (DJ)	DOI:10.1128/JB.00504–09	Dennis Dean, Virginia Tech; Wild-type (WT); Nif+
A. vinelandii strain	DJ2102	DOI:10.1016/bs.mie.2018.10.007	Dennis Dean, Virginia Tech; Strep-tagged WT NifD; Nif+
A. vinelandii strain	Δnif	Russell et al., 2024	Δ*nifHDK*::KanR + Δ*vnfDGK*::StrR + *anfD*::GenR; Nif-, Vnf-, Anf-
A. vinelandii strain	Anc^{AK029}	This paper	Δ*nifHDK*::$nifHDK^{Anc}$ (Strep-tagged NifD) + Δ*vnfDGK*::StrR + *anfD*::GenR; Nif+, Vnf-, Anf-
plasmid	pDB303	Dennis Dean	1.7-kbp EcoRI *A. vinelandii* fragment containing RifR determinant rpoB113
plasmid	$pAnc^{AK029}$	This paper	$nifHDK^{Anc}$ (Strep-tagged NifD) + 1000-bp *nifHDK* homology sequences , in pUC19 vector
primer	306_nifH_F	DOI:10.7554/eLife.85003	GCCGAACGTTCAAGTGGAAA
primer	307_nifH_R	DOI:10.7554/eLife.85003	AGAGCCAATCTGCCCTGTC
primer	308_nifD_F	DOI:10.7554/eLife.85003	CACCCGTTACCCGCATATGA
primer	309_nifD_R	DOI:10.7554/eLife.85003	ACTCATCTGTGAACGGCGTT
primer	310_nifK_F	DOI:10.7554/eLife.85003	GCTAACGCCGTTCACAGATG
primer	311_nifK_R	DOI:10.7554/eLife.85003	TCAGTTGGCCTTCGTCGTTG

References

1. Jimenez-Vicente E, Yang Z-Y, Martin del Campo JS, Cash VL, Seefeldt LC, Dean DR. 2019. The NifZ accessory protein has an equivalent function in maturation of both nitrogenase MoFe protein P-clusters. J Biol Chem 294:6204–6213.
